# Transvaginal ultrasound ovarian diathermy: sheep as an experimental model

**DOI:** 10.1186/1757-2215-5-1

**Published:** 2012-01-13

**Authors:** Anita M Pimentel, Danielle Kobayashi, Lucia M Kliemann, Renato Franjdlich, Edison Capp, Helena VE Corleta

**Affiliations:** 1 Programa de Pós-Graduação em Medicina: Ciências Médicas, Faculdade de Medicina, Universidade Federal do Rio Grande do Sul; 2 Departamento de Ginecologia e Obstetrícia da Faculdade de Medicina, Hospital de Clínicas de Porto Alegre, Universidade Federal do Rio Grande do Sul; 3 Departamento de Patologia, Faculdade de Medicina, Hospital de Clínicas de Porto Alegre, Universidade Federal do Rio Grande do Sul; 4 Gerar - Núcleo de Reprodução Humana do Hospital Moinhos de Vento, Porto Alegre, RS, Brazil

**Keywords:** transvaginal ovarian drilling, ovarian diathermy, sheep, polycystic ovary syndrome

## Abstract

**Background:**

Some techniques of transvaginal ovarian drilling have been previously described. Nevertheless a monopolar transvaginal ovarian cauterization, that use the expertise and safety of transvaginal puncture for oocyte captation seems to be an easier and feasible approach. The aim of this study was to develop a minimally invasive ovarian cauterization technique under transvaginal ultrasound control, and to evaluate the safety of the transvaginal ovarian monopolar cauterization, female sheep at reproductive age were used as an experimental model.

**Findings:**

An experimental study was performed in a university research center. Seventeen female sheep (15 Corriedale e 2 Suffolk) in reproductive age were submitted to transvaginal ovarian cauterization with a monopolar Valleylab Force 2 electrocautery. Macroscopic and microscopic lesions were assessed. Ovarian size were 1.31 cm^2 ^± 0,43 (Corriedale) and 3.41 cm^2 ^± 0,64 (Suffolk). From 30 ovaries from Corriedale sheep punctured, only 3 were cauterized, presenting macroscopic and typical microscopic lesion. In the Suffolk sheep group, only one ovary was cauterized. No lesion could be found in the needle path.

**Conclusions:**

This is the first experimental animal model described for ovarian cauterization needle guided by transvaginal ultrasound. The sheep does not seem to be the ideal animal model to study this technique. Another animal model, whose ovaries are better identified by transvaginal ultrasound should be sought for this technique, theoretically less invasive, before it could be offered safely to women with polycystic ovary syndrome.

## Findings

The polycystic ovarian syndrome (PCOS) is the endocrine-metabolic disorder that affects more women on reproductive age, with a prevalence of 5 to 10% [1]. This syndrome is characterized by anovulation, clinical or biochemical hyperandrogenism and ultrasound image showing several small ovarian follicles [2]. In addition, 60% of the patients are obese and several have insulin resistance [1].

Clomiphene citrate (CC) is the recommended first-line treatment for ovulation induction in PCOS patients [3]. However, around 20% of PCOS women are resistant to CC, requiring the second-line intervention: exogenous gonadotropins or laparoscopic ovarian surgery [3].

Ovulation induction with gonadotropins requires daily parenteral injections, intense monitoring of ovarian response, and is associated with increased occurrence of multiple pregnancy and ovarian hyperstimulation syndrome (OHSS) [4].

Laparoscopic ovarian surgery alone is as effective as gonadotropins to induce ovulation and has similar pregnancy rates. Laparoscopic drilling induces unifollicular ovulation with no risk of OHSS or high-order multiples [3]. Nonetheless, laparoscopic electrosurgical drilling requires hospital treatment, general anesthesia, and the risk of postoperative adhesions cannot be ignored general anesthesia [5,6].

Some techniques of transvaginal ovarian drilling have been described in elegant studies [7,8], nevertheless a monopolar transvaginal ovarian cauterization, that use the expertise and safety of transvaginal puncture for oocyte captation [9], seems to be an easier and feasible approach [10].

The aim of this study was to develop a minimally invasive ovarian cauterization technique under transvaginal ultrasound control. To evaluate the safety of the transvaginal ovarian monopolar cauterization, female sheep at reproductive age were used as an experimental model.

## Methods

Seventeen female sheep in reproductive age were included in this study. Ten days before the procedure, menstrual cycle in the sheep was induced and synchronized with the use of intravaginal pessaries with 50 mg of medroxyprogesterone acetate. Before the ovarian cauterization (48 h), the pessaries were removed and 300 to 600 IU of eCG (Novormon 5000, Intervet Schering-Plough Animal Health) was administered IM.

On the day of the procedure, the sheep was sedated with a combination of xylazine 2% and ketamine 10%. The animal was positioned in right lateral position and immobilized, the bladder emptied through catheterization. The pelvic structures were identified by transvaginal ultrasound. In the vaginal probe, a guide was attached guide and the puncture needle was inserted through this guide. The ultrasound equipment used was an Aloka 500 (Aloka, Tokyo, Japan) with vaginal probe of 6.5 MHz.

**Figure 1:**
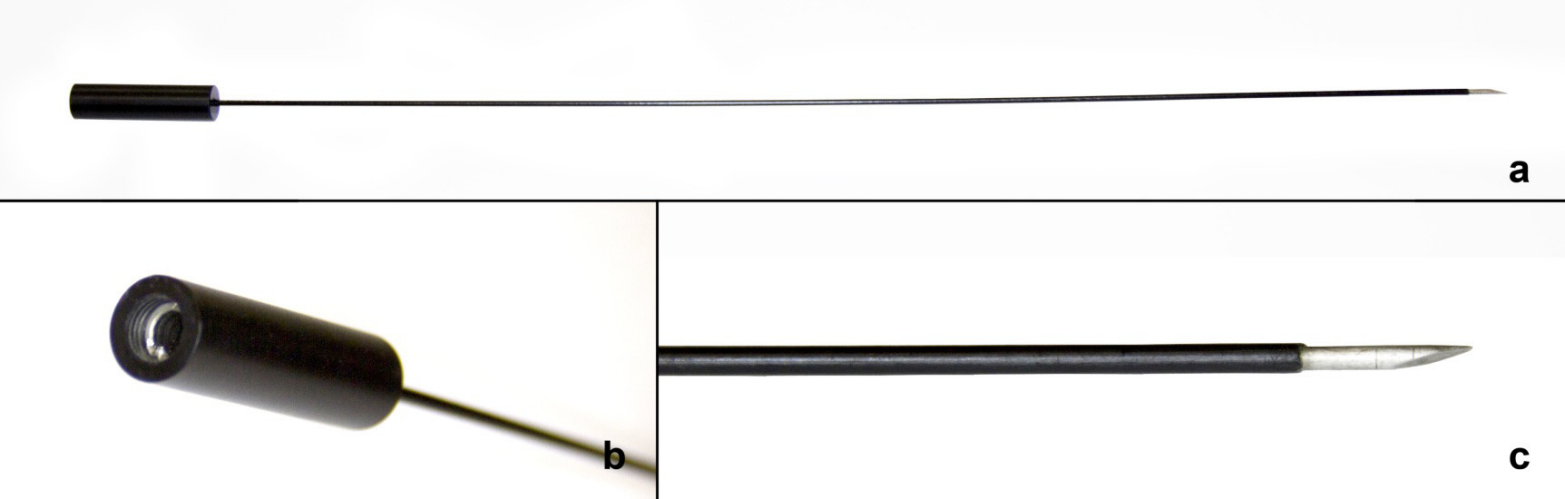
**Needle developed for transvaginal ovarian cauterization**: (a) the needle; (b) the conexion for eletrocautery; (c) the distal tip not insulated.

The needle for cauterization was exclusively developed for this study by Helena von Eye Corleta and manufactured in the Department of Biomedical Engineering of Hospital de Clínicas de Porto Alegre. It was made of stainless steel with 1.5 mm in diameter and 35 cm long, insulated throughout its length except for 3 mm distal. The proximal end was connected to the electrocautery (Figure 1).

The left ovary (LO) was punctured in four points and applied a voltage of 40 W for 5 s at each point, resulting in a total of 800 J (Joules) of thermal energy. In the right ovary (RO), the procedure was similar, with the same power for 10 s, resulting in a thermal energy of 1600 J. The electrocautery used was a Valleylab Force FX with monopolar coagulation (Valleylab, Boulder, USA).

Histopalogogical analysis was performed by experienced pathologist and changes caused by the puncture and cauterization were identified. Two days after the procedure, the sheep were slaughtered and a thorough inspection in the needle path, looking for lesions secondary to cauterization or puncture was performed.

Ovaries were collected, fixed in 10% formalin, sliced and submitted to the routine histological processing, dehydrating in alcohol, clearing in xylene, and paraffin impregnation. Slices (4 μm) were obtained and stained with hematoxylin-eosin technique.

This experiment was performed in accordance to the Brazilian College of Animal Experimentation (Colégio Brasileiro de Experimentação Animal - COBEA) and was approved by the Ethics Comittee of the Grupo de Pesquisa e Pós-Graduação do Hospital de Clínicas de Porto Alegre (#07113).

## Results

The procedure was performed in 15 Corriedale and 2 Suffolk female sheep. The weight of the animals and the size of the ovaries are shown in table 1.

**Table 1:** Sheep weight and ovaries sizes

Breed	Sheep weight (mean ± SD)	Ovaries sizes (mean ± SD)
Corriedale (n = 15)	34.5 kg ± 3.0	1.3 cm^2 ^± 0.4
Suffolk (n = 4)	105.0 ± kg ± 27.5	3.4 cm^2 ^± 0.6

**Figure 2:**
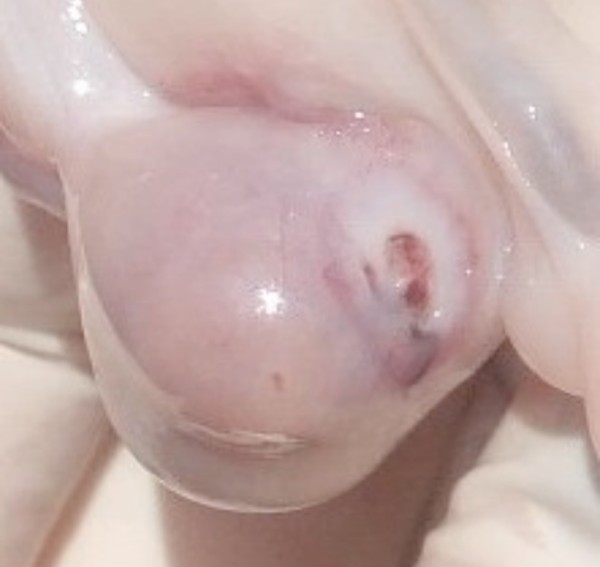
**Lesion in cauterized ovary of a Corriedale sheep (macroscopy)**.

**Figure 3:**
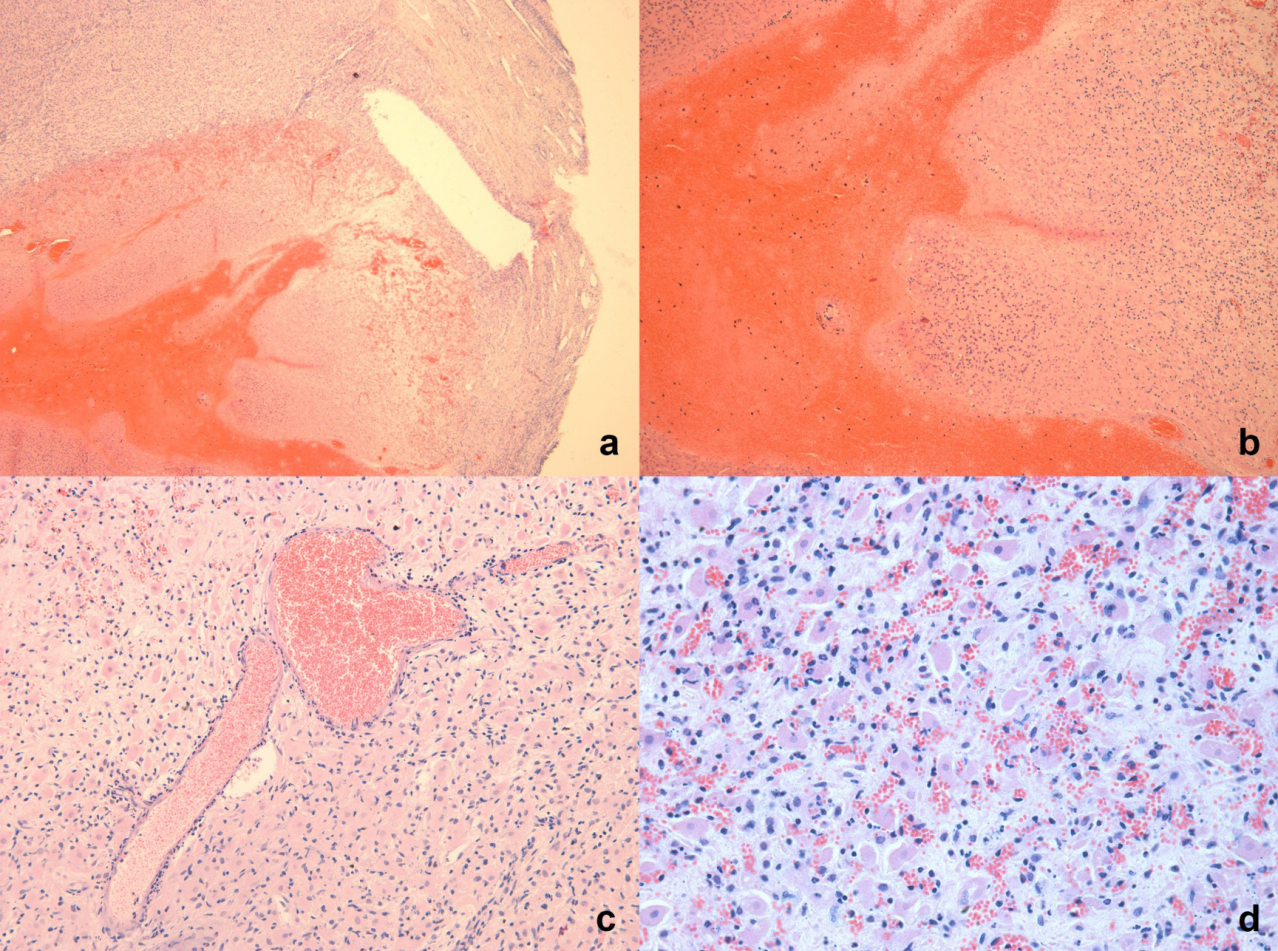
**Cauterization lesion (histology)**: (a) 40 X, general injury; (b) 100 X, bleeding: (c) 200 × blood vessels with neutrophiles: (d) 400 X, granulation tissue.

Of the 30 ovaries of Corriedale sheep, whose average size was 1.31 cm^3^, only 3 were affected by ovarian cauterization, presenting macroscopic (Figure 2) and typical microscopic lesion (Figure 3). In the Suffolk sheep group, only one ovary was cauterized.

Forty eight hours after cauterization, animals were sacrificed and injuries due to puncture or cauterization in the needle path were searched. Although the total thermal dose (n puncture × n second × Power W) delivered was 800 J (4 × 5 s × 40 W) on the right side and 1600 J (4 × 10 s × 40 W) on the left [5], no lesion could be found in the needle path.

## Discussion

Since 1990, less invasive methods for conducting and ovarian drilling [8,10,11] have been investigated, and a number of surgical procedures that destroy or remove ovarian tissue to restore ovulation in SOP patients have been described [12,13]. Although the surgical of new modalities have easy applicability and low cost with shorter hospital stay, the safety of the procedures remain to be defined [7].

In this study, the practicability and security of a transvaginal ovarian cauterization in a medium size animal (sheep), with the monopolar eletrocautery Valleylab was tested. The most frequently reported complication is alternate site burns due to high current density at an erroneously applied ground (return) electrode. This risk has been reduced by modern generators that are isolated from earth ground and have detectors that disable the machine and activate an alarm if the ground electrode circuit is faulty [14].

The main difficulty of the experiments was the correctly identification of the sheep ovaries. Ovarian size and antral follicles count are much smaller in the sheep than in cattle or humans. This may help explain the high variation in the gray-scale pixel values observed in the ultrasound image [15].

Even in the largest sheep, the ovaries (3.41 cm^3^) are much smaller than those found in women with PCOS, which have up to twice the size of the ovaries from ovulatory women (10 cm^3^) and are easily identified for ovarian puncture.

Besides the small size of the ovaries, another important factor contributing to the difficult identification of the gonads was the vaginal approach. In the sheep, follicles and uterus examination tend to have better results with rectal ultrasound [16]. However, for better simulation of the technique used in women, the transvaginal ultrasound and puncture, the same technique for oocyte retrieval in IVF procedures, was preferred.

In the four ovaries punctured and cauterized the histological lesions were characteristic with hemorrhage, necrosis and perivascular infiltration of neutrocytarian cells. Similar histological findings have been previously described 48 h after laser cauterization of sheep ovaries [17].

The positive aspect of this work is that no intraabdominal lesions secondary to puncture or cauterization were found in the needle path. It was surprising that after 2400 J of total thermal dose administered to the pelvis, no lesion was found. Probably, the area cauterized was a pelvic intra-abdominal fat, which showed no apparent tissue injury after 48 h. Adhesions were not identified, probably due to the short interval between the procedure and slaughter. A study about pelvic adhesions in sheep has been performed [18], however no experimental model about ovarian drilling complications could be found.

## Conclusions

This is the first experimental animal model described for ovarian cauterization needle guided by transvaginal ultrasound. The sheep does not seem to be the ideal animal model to study this technique: their ovaries are too small and too difficult to identify by transvaginal ultrasound. Another animal model, whose ovaries are better identified by transvaginal ultrasound should be sought for this technique, theoretically less invasive, before it could be offered safely to women with PCOS.

## Competing interests

The material contained in the manuscript has not been published or submitted elsewhere and it has not any vested conflict.

## Authors' contributions

AMP participated in the conception & design of study, data collection, data analysis and interpretation, surgery and imaging procedures, statistical analysis, manuscript preparation. DK participated in the data collection, data analysis and interpretation, statistical analysis, manuscript preparation. LMK participated in the data collection, data analysis and interpretation, manuscript preparation. RF participated in the data collection, data analysis and interpretation, surgery and imaging procedures. EC participated in the conception and design of study, data analysis and interpretation, statistical analysis, manuscript preparation. HvEC participated in the conception and design of study, data collection, data analysis and interpretation, surgery and imaging procedures, statistical analysis, manuscript preparation. All authors read and approved the final manuscript.
